# Pinostrobin from *Boesenbergia rotunda* attenuates oxidative stress and promotes functional recovery in rat model of sciatic nerve crush injury

**DOI:** 10.1590/1414-431X2023e12578

**Published:** 2023-02-27

**Authors:** R. Kongsui, S. Surapinit, T. Promsrisuk, S. Thongrong

**Affiliations:** 1 Division of Physiology, School of Medical Sciences, University of Phayao, Phayao, Thailand; 2 The Unit of Excellence in Translational Neurosciences Initiative, University of Phayao, Phayao, Thailand; 3 Department of Medical Technology, School of Allied Health Sciences, University of Phayao, Phayao, Thailand; 4 Division of Anatomy, School of Medical Sciences, University of Phayao, Phayao, Thailand

**Keywords:** Peripheral nerve injury, Boesenbergia rotunda, Pinostrobin, Oxidative stress, Antioxidant

## Abstract

Oxidative stress plays a role in the delay of peripheral nerve regeneration after injury. The accumulation of free radicals results in nerve tissue damage and dorsal root ganglion (DRG) neuronal death. Pinostrobin (PB) is one of the bioflavonoids from *Boesenbergia rotunda* and has been reported to possess antioxidant capacity and numerous pharmacological activities. Therefore, this study aimed to investigate the effects of PB on peripheral nerve regeneration after injury. Male Wistar rats were randomly divided into 5 groups including control, sham, sciatic nerve crush injury (SNC), SNC + 20 mg/kg PB, and SNC + 40 mg/kg PB. Nerve functional recovery was observed every 7 days. At the end of the study, the sciatic nerve and the DRG were collected for histological and biochemical analyses. PB treatment at doses of 20 and 40 mg/kg reduced oxidative stress by up-regulating endogenous glutathione. The reduced oxidative stress in PB-treated rats resulted in increased axon diameters, greater number of DRG neurons, and p-ERK1/2 expression in addition to faster functional recovery within 4 weeks compared to untreated SNC rats. The results indicated that PB diminished the oxidative stress-induced nerve injury. These effects should be considered in the treatment of peripheral nerve injury.

## Introduction

Peripheral nerve injury (PNI) is the most common traumatic injury. It occurs due to several causes such as sports accidents, traffic accidents, and medical operations (1). Unlike the central nervous system (CNS), the PNS has the ability of remodeling after injury (2). PNI affects quality of life through loss of both sensory and motor functions of the injured nerve (3,4). Therefore, a fast reinnervation of target organs such as skin and muscle is required to restore nerve function and improve the quality of life of patients.

Injury to peripheral nerves triggers an inflammatory process and induces the accumulation of free radicals such as reactive oxygen species (ROS) (5). ROS levels increase dramatically at the injured sciatic nerve and dorsal root ganglia (DRG). This increase plays a role in axon degeneration and induces DRG cell death (6). ROS such as the superoxide radical (^•^O_2_^−^) and the reactive hydroxyl radical (^•^OH) destroy the cell by oxidizing the lipids of the cell membrane. This process is called lipid peroxidation and produces cytotoxic agents, e.g., malondialdehyde (MDA), which cause oxidative stress (7). It is known that oxidative stress is one of the main causes of neuronal death and axon demyelination after nerve injury (8,9). The injured nerve has a mechanism to neutralize the effect of free radicals. However, the accumulation of free radicals can be uncontrolled and their elimination by the antioxidant defense system can be insufficient in response to oxidative stress after injury. This phenomenon weakens nerve repair and function recovery (10,11). Therefore, inhibition of oxidative stress using exogenous antioxidants may promote nerve reconstruction and restore nerve functions by preventing neuronal death and axonal demyelination after PNI.

*Boesenbergia rotunda* belongs to the Zingiberaceae family and is known as fingerroot or Krachai in Thai (12). *B. rotunda* is widely used as a cooking ingredient and in traditional medicine in Asia. Recently, researchers have reported that the rhizomes of *B. rotunda* contain many bioactive compounds such as terpenes, terpenoids, chalcone derivatives, and flavonoid derivatives (13). Pinostrobin (PB), or 5-hydroxy-7-methoxyflavanone, is a flavonoid found in the rhizome of *B. rotunda* (14). PB displays numerous pharmacological activities including anti-peptic ulcer, anti-cancer, anti-inflammatory, and antioxidant activities (15,16). An *in vitro* study showed that PB exerted a neuroprotective effect against beta-amyloid peptide-induced neurotoxicity in PC12 cells in a model of Alzheimer's disease (17). As is well known, oxidative stress and free radicals play a role in inhibiting nerve reconstruction and functional recovery after injury. Since PB possesses neuroprotective and antioxidant activities, this study was focused on the effect of PB on PNI using the sciatic nerve crush model. Because of its antioxidant properties, PB may be a new treatment strategy for nerve injury.

## Material and Methods

### Preparation of PB from *B. rotunda* rhizomes

Fresh rhizomes of *B. rotunda* were collected from Phayao province, Thailand. A plant specimen was authenticated by the Walai Rukhavej Botanical Research Institute, Mahasarakham University, Maha Sarakham, Thailand (Voucher No. S. Sedlak 19-1). The rhizomes were ground and dried to obtain an ethanolic crude extract (251 g). The extract was resuspended in methanol to yield a pale yellow methanol-insoluble solid (50 g). The obtained solid was further subjected to an open-column chromatography using silica gel (Arch.No. 7734, pore size 60 Å, particle size 70-230 mesh, Merck, USA) as an adsorbent. The column was eluted with a mixture of 40-80% dichloromethane-hexane under gradient separation. The fractions with similar patterns were combined based on thin-layer chromatography analysis and re-crystallized with methanol to yield pure forms of PB. Structural elucidation was performed by 13C- and 1H-NMR spectroscopy (Bruker Avance DRX500 Spectrometer, USA) and compared by nuclear magnetic resonance (NMR) spectra with the literature as follows: pinostrobin: 1H NMR (500 MHz, acetone-d6): 2.80 (dd, J=17.2, 3.0 Hz; 1H, H-3a), 3.06 (dd, J=17.2, 13.0 Hz; 1H, H-3b), 3.79 (s, 3H; -OCH3), 5.39 (dd, J=13.0, 3.0 Hz; 1H, H-2), 6.06 (m, 2H, H-6, H-8), 7.41 (m, 5H; H-2′, H-3′, H-3′, H-5′, H-6′). 13C NMR (150 MHz, acetone-d6): 43.1 (C-3), 55.7 (C-7 -OCH3), 79.2 (C-2), 94.3 (C-8), 95.1 (C-6), 103.1 (C-10), 126.1 (C-2′, C-3′, C-5′, C-6′), 128.9 (C-4′), 138.4 (C-1′), 162.8 (C-5), 164.1 (C-9), 167.9 (C-7), 195.8 (C-4).

### Animal model of sciatic nerve crush injury and PB administration

All animal procedures were approved by the Animal Ethics Committee of University of Phayao, Thailand (approval number 640104005). A total of 30 adult male Wistar rats weighing 200-220 g were purchased from Nomura Siam International Co., Ltd. (Thailand) and kept in a controlled room (25±2°C) with free access to food and water before the experiment. The rats were randomly divided into 5 groups (control, sham, sciatic nerve crush (SNC), SNC + 20 mg/kg of PB, and SNC + 40 mg/kg of PB, n=6 in each group). The SNC injury was performed as described previously (3). Briefly, a single dose of 50 mg/kg sodium pentobarbital was injected intraperitoneal to anesthetize the rats. A skin incision was made in the middle of the left thigh. The muscle layers were carefully split using blunt scissors at the intermuscular septum to disclose the sciatic nerve. The nerve was clamped with artery forceps (straight, 12 cm) for 30 s, 1 cm before the bifurcation. The sham rats received the same operation but without clamping. After operation, the skin was sutured with a nylon suture. In PB groups, the rats were treated with PB dissolved in 0.5% of carboxymethylcellulose sodium (Sigma-Aldrich, USA) once daily by oral gavage for 28 consecutive days after SNC.

### Sensory and motor function tests after SNC

The paw-withdrawal latency (PWL) was measured to determine the thermal hyperalgesia. Sensory test was performed on days 7, 14, 21, and 28 after nerve injury. The rats were acclimated to a non-heated plate for 10 min, and then their hind paws were thermally stimulated (50°C) at the plantar side. Threshold stimulation was recorded as PWL. PWL was recorded on the left paw three times with a 5-min interval between tests and the cut-off time of the experiment was 20 s (18). Motor functional recovery was assessed by walking track analysis on days 7, 14, 21, and 28 after operation. The hind paws of trained rats were soaked with water-soluble black ink. Then, the rats walked through a confined box (30×90×20 cm), with white paper (21×90 cm) placed on the bottom. The footprints were recorded to calculate the sciatic functional index (SFI) as proposed by Bain and colleagues (19). The motor functional recovery test was repeated three times for each rat.

### Determination of reduced glutathione and malondialdehyde levels

At the end of the experiment, the rats were sacrificed using CO_2_ at 30-70% volume displacement rate for 5 min. The distal stump of the sciatic nerve was removed and rinsed in ice-cold 0.9% sodium chloride. Then, the nerve was transferred to a lysis buffer containing 50 mM Tris-HCl (pH 7.4), 150 mM NaCl and 1% Triton X-100 to obtain the10% homogenate (w/v). The homogenized nerve was centrifuged at 9,279 *g* for 15 min at 4°C. The supernatant was collected and used to determine the antioxidant activity by the reduced glutathione (GSH) assay and lipid peroxidation by the malondialdehyde (MDA) assay.

Measurement of reduced GSH content followed the protocol of Sachett et al. (20). Briefly, 10 µL of supernatant was mixed in 160 µL of 1 M potassium phosphate buffer, 40 µL of 10 mM hydrogen peroxide (H_2_O_2_), and 40 µL of 6 mM reduced GSH in the dark for 30 min. Thereafter, the cocktail solution was transferred to a 96-well plate and mixed with 235 µL of 10 mM 5,5-dithio-bis-(2-nitrobenzoic acid) (DTNB) in the dark for 5 min. The absorbance of the solution was measured at 412 nm by a Cytation5 microplate reader (BioTek Instruments, Inc., USA). The result was provided as % of remaining GSH sulfhydryls.

The MDA assay was based on a previously published method (21). MDA reacts with thiobarbituric acid (TBA) to form thiobarbituric acid-reactive substances (TBARS). The results are reported as MDA detected by the Cytation5 microplate reader at 532 nm. A standard curve was generated with appropriate concentrations of 1,1.3,3-tetraethoxypropane (0.3-10 µmol/L). All biochemical assays were repeated three times.

### Histological analysis of sciatic nerve and DRG

The ipsilateral sciatic nerves about 5 mm distal to the crush site and L4-L6 DRG were collected on day 28 after injury. All tissues were fixed in 4% paraformaldehyde at 4°C for 48 h followed by immersion in 15% sucrose for 24 h and then 30% sucrose for 48 h for cryoprotection. Tissues were sectioned at 20-µm thickness using a cryostat (CM1950, Leica Microsystems, Inc., Germany). Sciatic nerve and DRG sections were used for hematoxylin & eosin (Sigma-Aldrich; Merck KGaA) staining. For immunofluorescence staining, DRG sections were stained with primary antibody against phospho-ERK1/ERK2 (Invitrogen; Cat# 44-680G, 1:100, USA) at 4°C overnight. Goat anti-rabbit Alexa Fluor 594 (Invitrogen; Cat# A-11012, 1:200) was used as secondary antibody in RT for 2 h. All images were obtained using the ECLIPSE Ni-U | Upright Microscopes (Nikon Corporation, Japan) and analyzed using NIS Elements imaging software version 5 (Nikon Corporation). The p-ERK1/2 fluorescence intensity was measured from the whole DRG and is reported as average fluorescence intensity after background subtraction.

### Statistical analysis

Statistical analyses were carried out using GraphPad Prism version 9 (GraphPad Software, Inc., USA). Data are reported as means±SE. A one-way ANOVA followed by a Tukey's *post hoc* test was used to analyze all data. P-values <0.05 were considered as significant.

## Results

### Effect of PB treatment on sciatic nerve oxidative stress after injury

**Figure 1:**
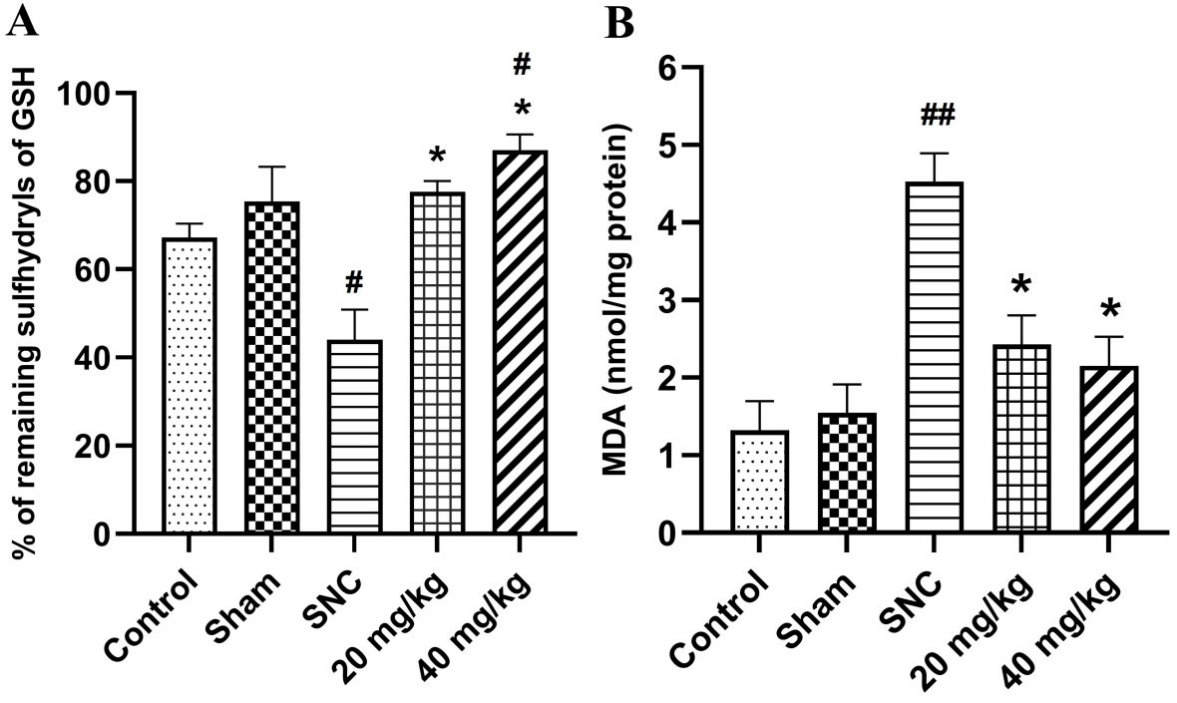
Effects of pinostrobin (PB) treatment (20 and 40 mg/kg) on reduced glutathione (GSH) sulfhydryls (**A**) and malondialdehyde (MDA) (**B**) levels after 28 days of sciatic nerve crush (SNC) injury. Data are reported as means±SE (n=6). *P<0.05 *vs* SNC group; ^#^P<0.05 and ^##^P<0.01 *vs* control group (ANOVA).

After 28 days of SNC, the administration of PB at doses of 20 and 40 mg/kg significantly increased GSH sulfhydryl molecules in sciatic nerve tissues compared with the SNC group (P<0.05) as shown in Figure 1A. The percent of GSH sulfhydryl was 77.6±2.4 and 87.0±3.6 in the 20 and 40 mg/kg-treated groups, respectively, whereas in the SNC group it was 44.0±6.8 percent. Moreover, PB at a dose of 40 mg/kg significantly (P<0.05) increased GSH sulfhydryl molecules in sciatic nerve tissues compared with the control group (67.2±3.2). MDA level was increased after SNC. Treatment with PB at doses of 20 and 40 mg/kg attenuated the level of MDA in the injured sciatic nerve. Oral administration of 20 and 40 mg/kg of PB caused a significant decrease in MDA level compared to the SNC group (P<0.05). The MDA levels were 4.52±0.36 nmol/mg protein in the SNC group, 2.43±0.37 nmol/mg protein at 20 mg/kg, and 2.15±0.40 nmol/mg protein at 40 mg/kg (Figure 1B). However, there was no significant difference in MDA from the control (1.32±0.38 nmol/mg protein) and PB-treated groups.

### Effect of PB treatment on histological alteration after sciatic nerve injury

**Figure 2:**
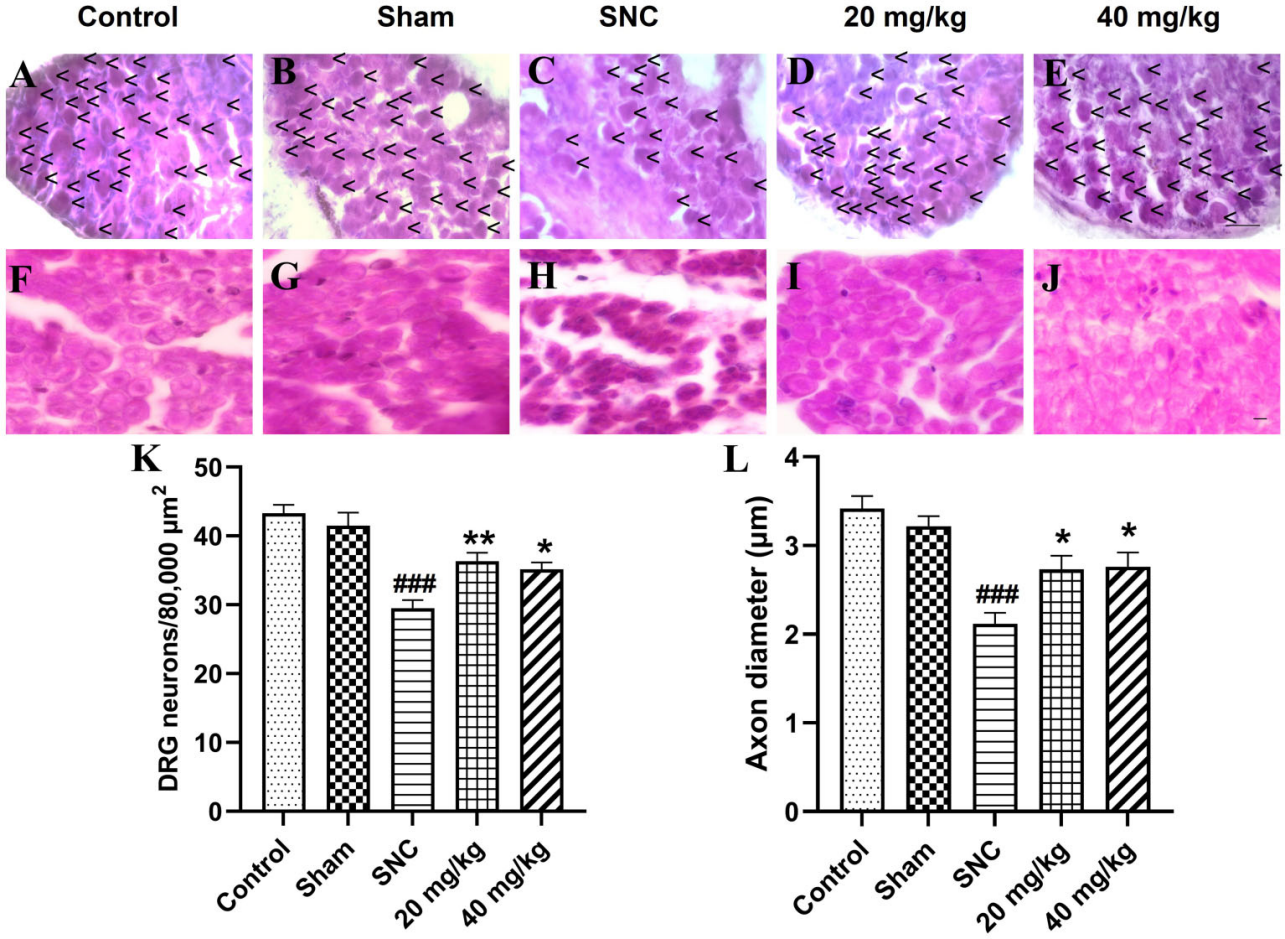
Effects of pinostrobin (PB) treatment (20 and 40 mg/kg) on dorsal root ganglion (DRG) neurons and axon diameters on day 28 after sciatic nerve crush (SNC). **A**-**E**, H&E staining of DRG neurons (arrow heads) (scale bar=50 µm) and (**F**-**J**) histological analysis of axons (scale bar=5 µm). **K**, Quantitative analyses of number of DRG neurons and (**L**) axon diameter. Data are reported as means±SE (n=6). *P<0.05, **P<0.01 *vs* SNC group and ^###^P<0.001*vs* control group (ANOVA).

Histopathological analyses of DRG and sciatic nerve were performed on day 28 after operation. H&E staining of ipsilateral L4-L6 DRG and sciatic nerve are shown in Figure 2A-J). Oral administration of PB at doses of 20 and 40 mg/kg caused a significant increase in the number of DRG neurons compared to the SNC group (P<0.01 and P<0.05, respectively). The number of DRG per area was 43.32±1.21 in the control group, 29.50±1.18 in the SNC group, 36.37±1.21 at 20 mg/kg, and 35.16±0.99 at 40 mg/kg (Figure 2K). Nerve regeneration was analyzed by axon diameter measurement. On day 28 after nerve injury, the axon diameters were decreased in all injured rats. Axon diameters were 3.42±0.14 μm in the control group, 2.11±0.12 μm in the SNC group, 2.73±0.15 μm at 20 mg/kg, and 2.76±0.16 μm at 40 mg/kg. The oral gavage of PB at doses of 20 and 40 mg/kg had the effect of increasing the axon diameter compared to the SNC group (P<0.05), as shown in Figure 2L.

### Effect of PB treatment on p-ERK1/2 expression in L4-L6 DRG after sciatic nerve injury

**Figure 3:**
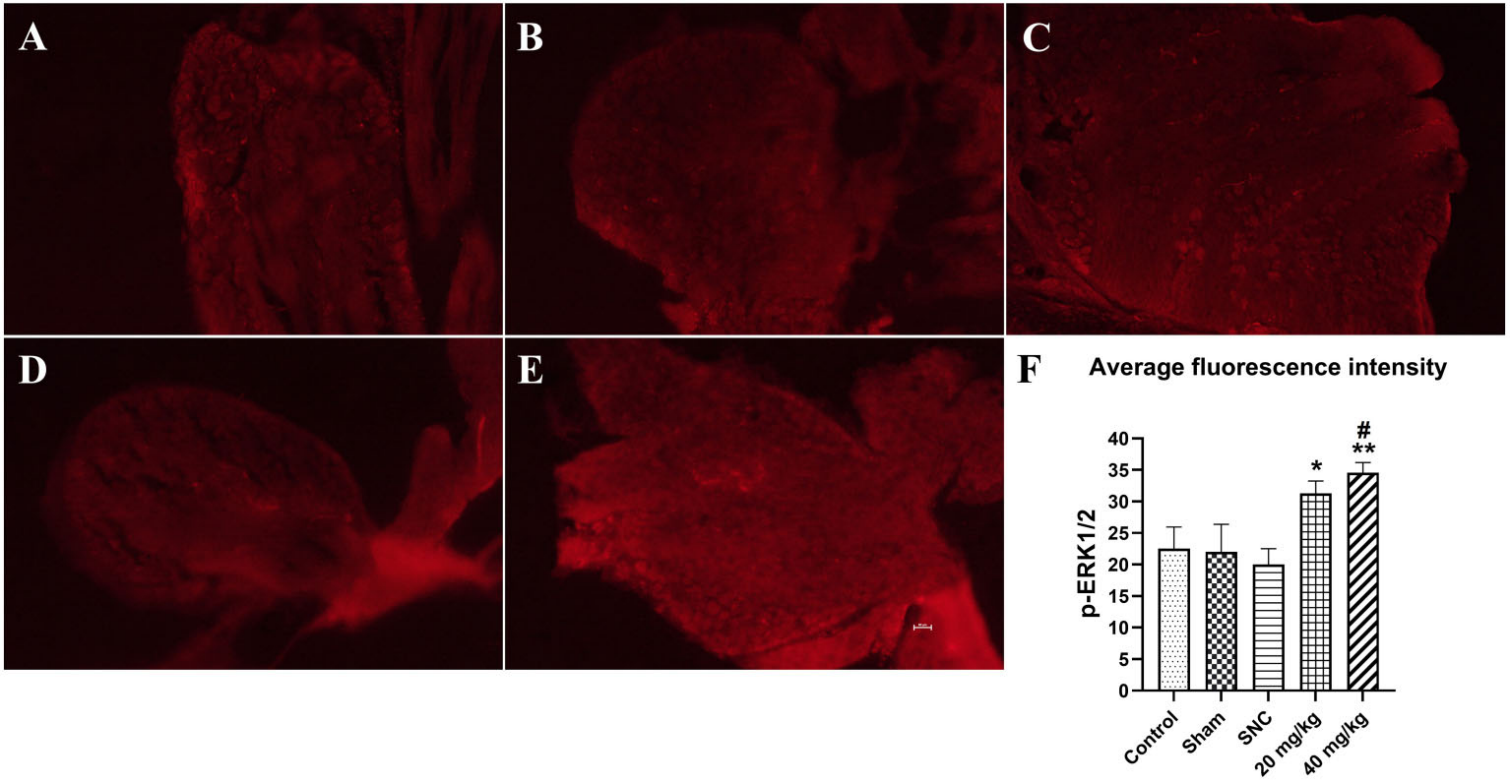
Effects of pinostrobin (PB) treatment (20 and 40 mg/kg) on p-ERK1/2 average fluorescence intensity in dorsal root ganglion (DRG) after sciatic nerve crush (SNC) at 28 days. (**A**) Control, (**B**) Sham, (**C**) SNC, (**D**) 20 mg/kg PB, and (**E**) 40 mg/kg PB (scale bar=50 µm). **F**, Quantitative analysis of p-ERK1/2 average fluorescence intensity in DRG on day 28 after SNC. Data are reported as means±SE (n=6). *P<0.05, **P<0.01 *vs* SNC group and ^#^P<0.05 *vs* control group (ANOVA). p-ERK1/2: phosphorylated extracellular signal-regulated kinase1/2.

Phosphorylated extracellular signal-regulated kinase1/2 (p-ERK1/2) has been reported as an activation signal for survival of neurons (22). In this study, administration of PB after SNC promoted the survival of DRG neurons, which may involve p-ERK1/2 activation. On day 28 after SNC, PB administration at doses of 20 and 40 mg/kg caused a significant increase in p-ERK1/2 average fluorescence intensity compared to the SNC group (P<0.05 and P<0.01, respectively), as reported in Figure 3.

### Effect of PB administration on functional restoration after SNC

**Figure 4:**
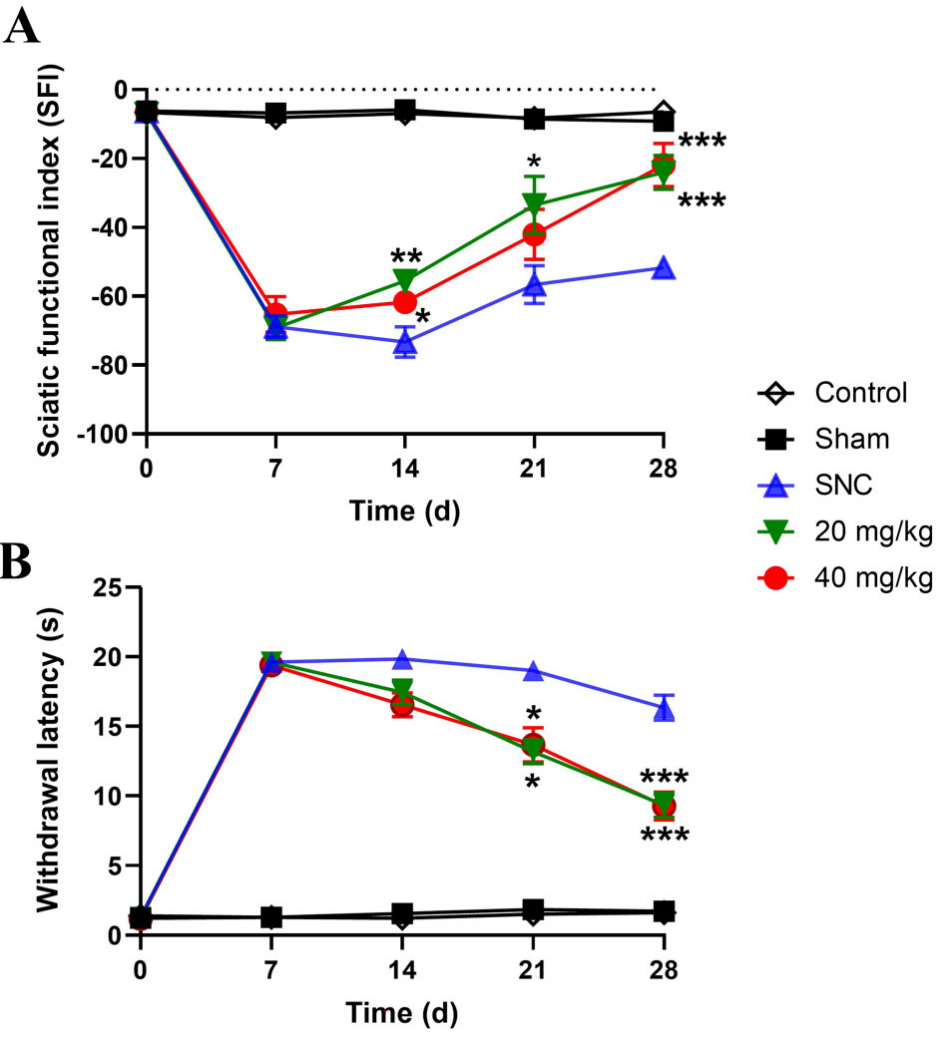
Effects of pinostrobin (PB) treatment (20 and 40 mg/kg) on motor (**A**) and sensory (**B**) functional recovery after sciatic nerve crush (SNC). Data are reported as means±SE (n=6). *P<0.05, **P<0.01, and ***P<0.001 *vs* SNC group (ANOVA).

Prior to SNC, baseline motor and sensory functions did not differ between the rats. Suddenly after nerve injury, all rats exhibited motor and sensory function deficits, as shown in Figure 4. Sciatic functional index, as represented by motor function recovery, was significantly improved in rats treated with PB at doses of 20 and 40 mg/kg on day 14 after SNC (P<0.01 and P<0.05, respectively). Motor function in PB-treated groups improved significantly with time after nerve injury. PB-administrated rats at doses of 20 and 40 mg/kg showed significant differences in motor function recovery compared to SNC rats on day 28 after nerve injury (P<0.001) (Figure 4A). Sensory function recovery was assessed by PWL test using heat stimulation. PB administration at doses of 20 and 40 mg/kg caused a significant improvement in sensory function compared to the SNC group on day 21 after nerve injury (P<0.05) and an even greater improvement on day 28 after nerve injury compared with the SNC group (P<0.001) (Figure 4B).

## Discussion

In this study, we investigated the antioxidant and therapeutic effects of PB from *B. rotunda* at doses of 20 and 40 mg/kg on SNC injury in rats. The results demonstrated that PB administration increased antioxidant activity and attenuated oxidative stress level after sciatic nerve injury. These effects corresponded with the improved axon remyelination and functional recovery. Soon after nerve injury, injured nerves exhibited activation of lipid peroxidation as indicated by the elevation of MDA level. MDA, a biomarker product of polyunsaturated fatty acid, plays a role in the demyelination of the injured nerve (23). Not only injured nerves but also intact DRG are affected by overproduction of free radicals after nerve damage leading to apoptotic death of DRG neurons (24). Our results illustrated that oral administration of PB for 28 days could attenuate MDA levels of sciatic nerve tissues after injury. In the CNS, it has been shown that administration of PB could prevent neurotoxin-induced death of dopaminergic neurons via suppression of MDA and ROS production (25). Moreover, we found that PB treatment elevated antioxidant activity of sciatic nerve tissues. The % of remaining GSH sulfhydryl in PB-treated rats was significantly higher than in the SNC rats. Evidence has shown that PB could increase the activities of antioxidant enzymes including catalase, superoxide dismutases, and glutathione peroxidases in an *in vitro* experiment (26). These results clearly suggested that the 28-day administration of PB after peripheral nerve injury could reduce oxidative stress by increasing antioxidant activity.

In the present study, it was shown that SNC injury created histological changes including changes in myelinated axon diameter and number of DRG neurons. A previous experiment has shown that injured sciatic nerve tissues display high oxidative stress and proinflammatory cytokine expressions, resulting in myelin sheath degeneration and loss of nerve fibers (5). Our finding showed that the axon diameters were decreased after sciatic nerve injury and were related to the reduction of motor function. After injury, the nerve establishes axon regrowth and remyelination to connect the nerve to target organs such as muscles and skin. Muscular reinnervation is associated with axon remyelination in motor functional recovery (27).

It is well known that oxidative stress plays a role in the destruction of axons and retards motor restoration after nerve injury (28). In the present study, oral administration of PB for 28 days resulted in a significant increase in axon diameters compared with SNC alone. Moreover, PB could improve motor functional recovery as indicated by a significant increase in sciatic functional index within 14 days after nerve lesion. Based on our results, PB treatment elevated the GSH level in sciatic nerve tissues, and GSH has been reported to play a role in the neutral system to protect nerve components from oxidative stress (29). Therefore, high GSH level in injured sciatic nerves may increase axon diameters and improve the motor function recovery after nerve injury.

Additionally, nerve damage also causes sensory loss (3). Our results showed that PB treatments after injury to sciatic nerve could improve the number of DRG neurons in L4-L6 DRG and restore sensory function within 21 days. Previous studies have shown that injury to peripheral nerves causes the loss of motor and sensory neurons. Sensory neurons in DRG are more vulnerable than spinal cord motor neurons (30,31).

Apoptotic death of DRG neurons occurs after the first week of nerve injury (32). Therefore, a reduction in the number of DRG neurons can reduce the sensory functional recovery after nerve injury (33). PB prevented the loss of DRG neurons and accelerated sensory functional recovery, which is likely associated with increased antioxidant levels after nerve injury. Antioxidants increase can mediate the intracellular signaling pathways such as ERK/Nrf2 (extracellular signal-regulated protein kinase/nuclear factor erythroid 2-related factor 2), an antioxidant defense system for suppressing oxidative stress-induced neuronal death (34,35). Previous experiments have found that phosphorylated ERK (p-ERK) was increased in DRG and proximal nerve stump after SNC injury (36,37). The increase in p-ERK has been known to promote neurite elongation and axon regeneration (38,39). The present results showed that oral administration of PB increased the number of DRG neurons compared to the non-treated rats after sciatic nerve crush injury. The neuroprotective effect was likely due to antioxidant activation of p-ERK1/2. Higher number of DRG neurons in PB-treated rats was associated with higher p-ERK1/2 expression compared with the SNC group. These results suggested that the increase in DRG neurons was regulated by p-ERK1/2, as a survival molecule (22,40). Taken together, these studies suggest that PB enhanced antioxidant levels to promote nerve remyelination and protect DRG neurons from apoptotic death, which resulted in faster functional recovery after SNC injury, and the mechanism likely involves the activation of Erk signaling pathway.

### Conclusion

In conclusion, administration of PB at doses of 20 and 40 mg/kg after SNC injury can promote nerve axon remyelination and survival of DRG neurons. However, there was no difference between the low and high doses of PB on peripheral nerve injury. The therapeutic effects of PB were through its antioxidant activities as indicated by high GSH level and low MDA level. The neuroprotective effect against SNC-induced DRG neuronal death is most likely due to PB-activated pERK1/2 expression. These results indicated that PB could be considered as a treatment for peripheral nerve injury. Our ongoing study is focused on the effects of different doses of PB on the peripheral and central nervous systems.
